
JOURNAL INFORMATION
==============================
NLM Title Abbreviation: J Diabetes Investig
Journal ID: J Diabetes Investig
NLM Journal ID: 101520702
Journal ID: JDI

Journal of Diabetes Investigation

ISSN: 2040-1116
EISSN: 2040-1124
Publisher: Wiley

ARTICLE INFORMATION
==============================
PMCID: PMC11474720
DOI: 10.1111/jdi.14248
Article ID: JDI14248
Article version: 1
Subjects: Issue Information, Issue Information

Issue Information

Electronic publication date: 2024 Jun 1
Publication date: 2024 Jun
Volume: 15
Issue: 6
Issue title: β‐cell and incretin therapy
First page: 645
Last page: 646
Copyright: © 2024 The Authors. Journal of Diabetes Investigation published by Asian Association for the Study of Diabetes (AASD) and John Wiley & Sons Australia, Ltd
License: This is an open access article under the terms of the http://creativecommons.org/licenses/by/4.0/ License, which permits use, distribution and reproduction in any medium, provided the original work is properly cited.
License URL: https://creativecommons.org/licenses/by/4.0/

Figure count: 0
Table count: 0
Page count: 5

source-schema-version-number: 2.0
cover-date: June 2024
details-of-publishers-convertor: Converter:WILEY_ML3GV2_TO_JATSPMC version:6.4.9 mode:remove_FC converted:15.10.2024

==============================

### JDI

Volume 15 | Issue 6
June 2024

**Journal of Diabetes Investigation**
Official Journal of the Asian Association for the Study of Diabetes

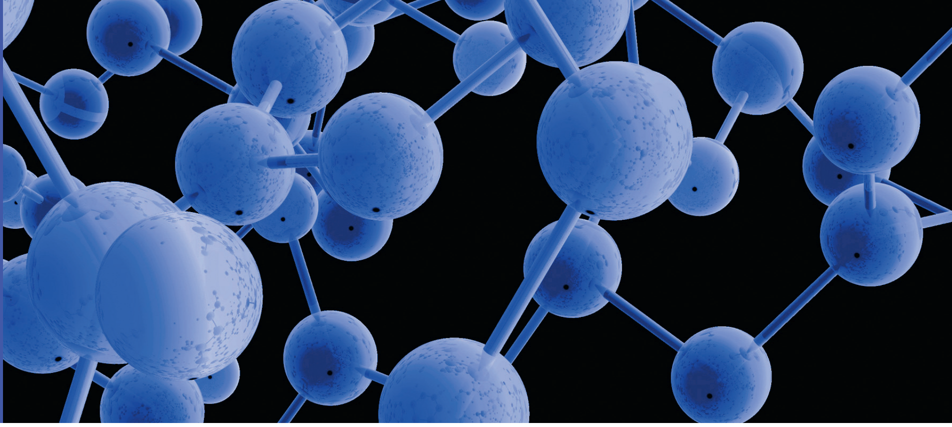

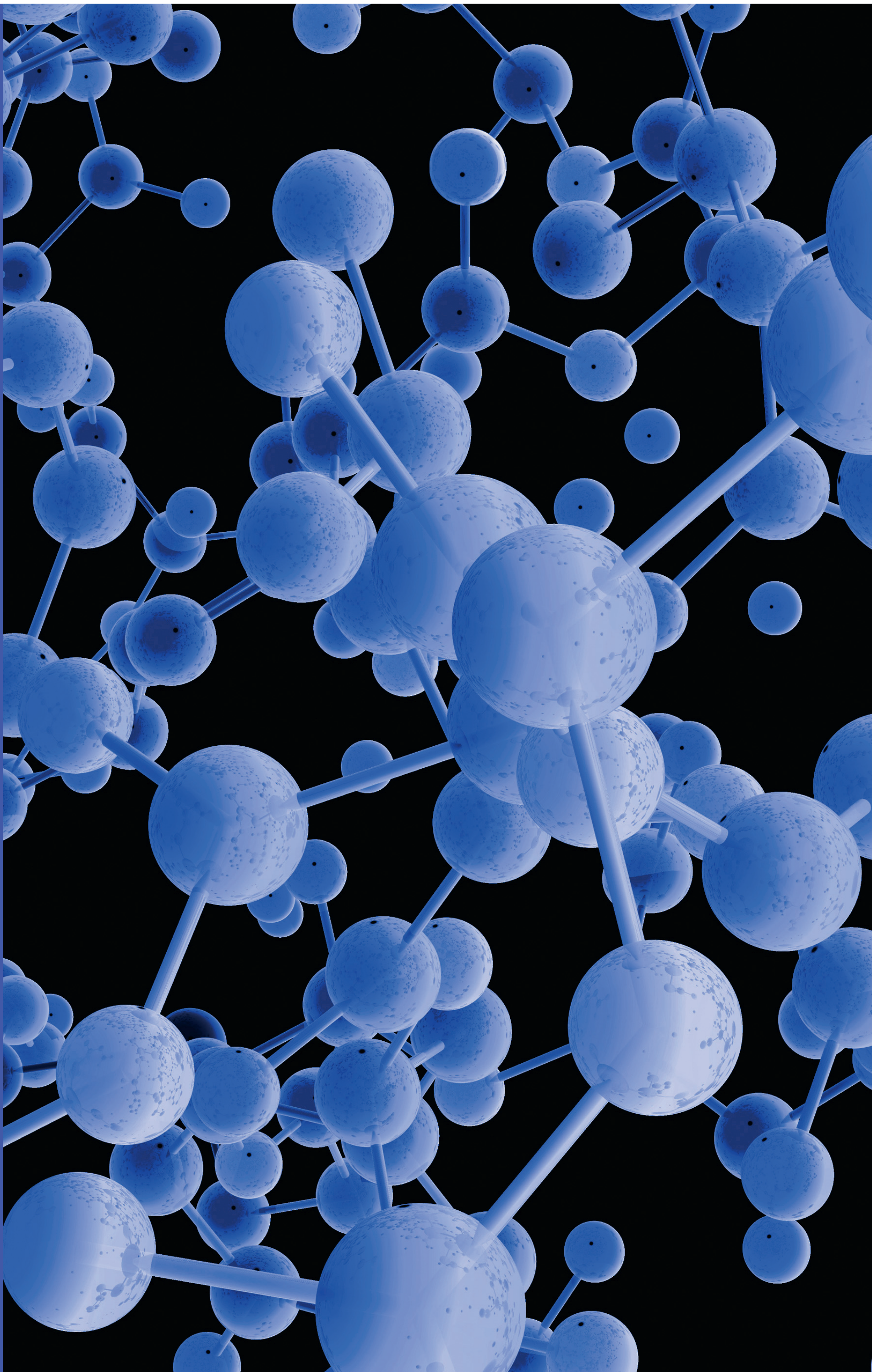

LBW and *RETN* SNP influence T2DM risk

THE ASIAN ASSOCIATION FOR THE STUDY OF DIABETES
WILEY

### JDI Journal of Diabetes Investigation

Open access

Official Journal of the Asian Association for the Study of Diabetes

**Editor-in-Chief**
Nigishi Hotta, *Japan Organization of Occupational Health and Safety, Chubu Rosai Hospital, Nagoya, Japan*

**Co-Editor**
Domenico Accili, *Columbia University, New York, USA*

**Expert Editor**
Bo Ahren, *Lund, Sweden*
Young Min Cho, *Seoul, Korea*
Sean Dinneen, *Galway, Ireland*
Marc Gilbert, *Montpelier, France*
Limei Liu, *Shanghai, China*
Ronald Ma, *Hong Kong, China*
Louis Philipson, *Chicago, USA*
Wayne H-H Sheu, *Taichung, Taiwan*
Bernard Thorens, *Lausanne, Switzerland*
Daisuke Yabe, *Kyoto, Japan*

**Editorial Board**
Eiichi Araki, *Kumamoto, Japan*
Xiaoping Chen, *Beijing, China*
Lee-Ming Chuang, *Taipei, Taiwan*
Masakazu Haneda, *Asahikawa, Japan*
Larry Low-Tone Ho, *Taipei, Taiwan*
Tianpei Hong, *Beijing, China*
Akihisa Imagawa, *Osaka, Japan*
Nobuya Inagaki, *Kyoto, Japan*
Linong Ji, *Beijing, China*
Weiping Jia, *Shanghai, China*
Yi-Der Jiang, *Taipei, Taiwan*
Takashi Kadowaki, *Tokyo, Japan*
Kohei Kaku, *Kurashiki, Japan*
Keizo Kanasaki, *Izumo, Japan*
Doo-Man Kim, *Seoul, Korea*
Karen Lam, *Hong Kong, China*
Hong Kyu Lee, *Gyeonggi-do, Korea*
In-Kyu Lee, *Daegu, Korea*
Moon-Kyu Lee, *Seoul, Korea*
Jiro Nakamura, *Nagakute, Japan*
Kishio Nanjo, *Wakayama, Japan*
Wataru Ogawa, *Kobe, Japan*
Yutaka Seino, *Osaka, Japan*
Shyi-Jang Shin, *Kaohsiung, Taiwan*
Hirohito Sone, *Niigata, Japan*
Kathryn Tan, *Hong Kong, China*
Yukio Tanizawa, *Ube, Japan*
Kohjiro Ueki, *Tokyo, Japan*
Jun Wada, *Okayama, Japan*
Hirotaka Watada, *Tokyo, Japan*
Jianping Weng, *Guangzhou, China*
Kyu-Chang Won, *Daegu, Korea*
Soroku Yagihashi, *Hirosaki, Japan*
Hiroshi Yamamoto, *Kanazawa, Japan*
Toshimasa Yamauchi, *Tokyo, Japan*
Wenying Yang, *Beijing, China*
Kazuki Yasuda, *Tokyo, Japan*
Koutaro Yokote, *Chiba, Japan*
Kun-Ho Yoon, *Seoul, Korea*
Dalong Zhu, *Nanjing, China*

**International Editorial Board**
Michael A. Brownlee, *New York, USA*
Subrata Chakrabarti, *Ontario, Canada*
Mark E. Cooper, *Prahran, Australia*
Philippe Froguel, *London, UK*
Wilfred Fujimoto, *Hawaii, USA*
Michael S. German, *San Francisco, USA*
Andrew Hattersley, *Exeter, UK*
Jean-Claude Henquin, *Brussels, Belgium*
Jens Juul Holst, *Copenhagen, Denmark*
Frank B. Hu, *Boston, USA*
C. Ronald Kahn, *Boston, USA*
George L. King, *Boston, USA*
Rayaz A. Malik, *Manchester, UK*
Michael Mauer, *Minneapolis, USA*
Carl Erik Mogensen, *Aarhus, Denmark*
Steven E. Shoelson, *Boston, USA*
Jay S. Skyler, *Miami, USA*
Paul Zimmet, *Melbourne, Australia*

**Assistant Editorial Board**
Tien-Jyun Chang, *Taipei, Taiwan*
Yoshiyuki Hamamoto, *Osaka, Japan*
Norio Harada, *Kyoto, Japan*
Yi-Jen Hung, *Taipei, Taiwan*
Chi-Min Hwu, *Taipei, Taiwan*
Hideki Kamiya, *Aichi, Japan*
Dae Jung Kim, *Suwon, Korea*
Seung-Hwan Lee, *Seoul, Korea*
Hung-Yuan Li, *Taipei, Taiwan*
Takashi Matsuzaka, *Tsukuba, Japan*
Keiko Naruse, *Aichi, Japan*
Takeshi Nishikawa, *Kumamoto, Japan*
Eung-Jung Rhee, *Seoul, Korea*
Yusuke Seino, *Aichi, Japan*
Hironori Waki, *Tokyo, Japan*

**Aims and Scope:** *Journal of Diabetes Investigation* is your core diabetes journal from Asia; the official journal of the Asian Association for the Study of Diabetes (AASD). The journal publishes original research, country reports, commentaries, reviews, mini-reviews, case reports, letters, as well as editorials and news. The journal embraces clinical and experimental research in diabetes and related areas. This includes aspects of prevention, treatment, as well as molecular aspects and pathophysiology. Translational research focused on the exchange of ideas between clinicians and researchers is also welcome.

**Cover:** Insulin molecule © Pasieka/Science Photo Library

This journal is available online at Wiley Online Library. Visit wileyonlinelibrary.com to search the articles and register for table of contents and e-mail alerts.

**For submission instructions, and all other information visit wileyonlinelibrary.com/journal/jdi**

JDI.JEB.Jun24

**Open Access and Copyright**
All articles published by *Journal of Diabetes Investigation* are fully open access: immediately freely available to read, download and share. All *Journal of Diabetes Investigation* articles accepted from 24 September 2013 are published under the terms of the Creative Commons Attribution 4.0 Unported (CC BY 4.0) license, which permits use, distribution and reproduction in any medium, provided the original work is properly cited. Articles accepted before this date were published under the agreement as stated in the final article. Copyright on any *Journal of Diabetes Investigation* article accepted after 24 September 2013, the date on which *Journal of Diabetes Investigation* became an open access journal, is retained by the author(s). Authors grant to *Journal of Diabetes Investigation* and Wiley a license to publish the article and for Wiley to identify itself as the original publisher. Authors also grant any third party the right to use the article freely as long as its integrity is maintained and its original authors, citation details and publisher are identified. Further information about open access licenses and copyright can be found at: https://authorservices.wiley.com/author-resources/Journal-Authors/licensing/licensing-info-faqs.html#4. Information on the licenses used by Journal of Diabetes Investigation can be found at: wileyonlinelibrary.com/journal/jdi

**Disclaimer**
The Publisher, the Asian Association for the Study of Diabetes, and Editors cannot be held responsible for any errors in or any consequences arising from the use of information contained in this journal. The views and opinions expressed do not necessarily reflect those of the Publisher, the Asian Association for the Study of Diabetes or Editors, neither does the publication of advertisements constitute any endorsement by the Publisher, the Asian Association for the Study of Diabetes, Editors, or Authors, of the products advertised.

Wiley Open Access articles posted to repositories or websites are without warranty from Wiley of any kind, either express or implied, including, but not limited to, warranties of merchantability, fitness for a particular purpose, or non-infringement. To the fullest extent permitted by law Wiley disclaims all liability for any loss or damage arising out of, or in connection, with the use of or inability to use the content.

JDI.PI.Jun24

VOLUME 15 – ISSUE 6 – JUNE 2024

### Journal of Diabetes Investigation

Open access

### Special Issue: β-cell and incretin therapy

Issue edited by: Professor Kun-Ho Yoon

### Reviews

**Glucose metabolism partially regulates β-cell function through epigenomic changes**
Yong Kyung Kim, Kyu Chang Won, and Lori Sussel **649**

**Role of β-cell autophagy in β-cell physiology and the development of diabetes**
Xaviera Riani Yasasilka and Myung-Shik Lee **656**

**Pancreatic beta-cell mass and function and therapeutic implications of using antidiabetic medications in type 2 diabetes**
Joon Ho Moon, Hun Jee Choe, and Soo Lim **669**

### Original Articles

**Mitochondrial ALDH2 improves ß-cell survival and function against doxorubicin-induced apoptosis by targeting CK2 signaling**
Udayakumar Karunakaran, Eun Yeong Ha, Suma Elumalai, Kyu Chang Won, and Jun Sung Moon **684**

**Long-term efficacy of encapsulated xenogeneic islet transplantation: Impact of encapsulation techniques and donor genetic traits**
Heon-Seok Park, Eun Young Lee, Young-Hye You, Marie Rhee, Jong-Min Kim, Seong-Soo Hwang, and Poong-Yeon Lee **693**

### Regular Articles

### Commentary

**Effects of dipeptidyl peptidase-4 inhibition *in vivo*: Dipeptidyl peptidase-4 inhibitor/gut microbiome crosstalk suggests novel therapeutic options for diabetes management**
Takuma Yasuda, Norio Harada, and Nobuya Inagaki **704**

### Update

**Interplay of cardiovascular health and diabetes: Insights into weight management and risk reduction**
Chih-Yao Hsu and Hung-Yuan Li **707**

### Original Articles

#### Basic Science and Research

**High glucose promotes atherosclerosis by regulating miRNA let7d-5p level**
Hua Wang, Fentao Liu, Wenyu Zhao, Yiting Guo, Peipei Mai, Songfeng Zhao, Zhiguo Wen, Jie Su, Xuan Li, Yunlong Wang, and Yanfang Zhang **711**

#### Clinical Science and Care

**Interaction effect between low birthweight and resistin gene rs1862513 variant on insulin resistance and type 2 diabetes mellitus in adulthood: Toon Genome Study**
Ayaka Yoshida, Yasunori Takata, Yasuharu Tabara, Koutatsu Maruyama, Shota Inoue, Haruhiko Osawa, and Takashi Sugiyama **725**

**Simplified electrophysiological approach combining a point-of-care nerve conduction device and an electrocardiogram produces an accurate diagnosis of diabetic polyneuropathy**
Yusuke Hayashi, Tatsuhito Himeno, Yuka Shibata, Nobuhiro Hirai, Yuriko Asada-Yamada, Sachiko Sasajima, Emi Asano-Hayami, Mikio Motegi, Saeko Asano, Makoto Kato, Hiromi Nakai-Shimoda, Hiroya Tani, Emiri Miura-Yura, Yoshiaki Morishita, Masaki Kondo, Shin Tsunekawa, Takayuki Nakayama, Jiro Nakamura, and Hideki Kamiya **736**

**Performance of different machine learning algorithms in identifying undiagnosed diabetes based on nonlaboratory parameters and the influence of muscle strength: A cross-sectional study**
Ying Xu, Shanhu Qiu, Jinli Ye, Dan Chen, Donglei Wang, Xiaoying Zhou, and Zilin Sun **743**

**Maternal birth weight as an indicator of early and late gestational diabetes mellitus: The Japan Environment and Children's Study**
Kazuma Tagami, Noriyuki Iwama, Hirotaka Hamada, Hasumi Tomita, Rie Kudo, Natsumi Kumagai, Hongxin Wang, Seiya Izumi, Zen Watanabe, Mami Ishikuro, Taku Obara, Nozomi Tatsuta, Hirohito Metoki, Chiharu Ota, Takashi Sugiyama, Shinichi Kuriyama, Takahiro Arima, Nobuo Yaegashi, and Masatoshi Saito, The Japan Environment and Children's Study Group **751**

**Association between serum uric acid-to-high-density lipoprotein cholesterol ratio and insulin resistance in an American population: A population-based analysis**
Xiaohai Zhou and Jing Xu **762**

**Health impacts of new-onset diabetes in women post-gestational diabetes mellitus: Insights from Hong Kong's territory-wide data**
Xinge Zhang, Hongjiang Wu, Eric SH Lau, Baoqi Fan, Kit Ying Tsoi, Claudia HT Tam, Aimin Yang, Mai Shi, Elaine Chow, Alice PS Kong, Juliana CN Chan, Wing Hung Tam, Andrea OY Luk, and Ronald CW Ma **772**

### Case Report

**Corticosteroid-triggered acute skeletal muscle loss in lipodystrophy: A case report**
Takayoshi Sasako, Ken Suzuki, Sara Odawara, Hirotsugu Suwanai, Naoko Akuta, Naoto Kubota, Kohjiro Ueki, Takashi Kadowaki, and Toshimasa Yamauchi **782**

**Fulminant type 1 diabetes, an underrecognized and unique subtype of type 1 diabetes: A case series from Singapore**
Sarah Ying Tse Tan, Suresh Rama Chandran, Jielin Yew, Andy Jun-Wei Wong, and Daphne Su-Lyn Gardner **786**

### Letter To The Editor

**Comment on a recent article titled 'Hepatocyte nuclear factor 1B deletion, but not intragenic mutation, might be more susceptible to hypomagnesemia'**
Rhian Clissold, Coralie Bingham, and Andrew Hattersley **790**

### Corrigendum

**Corrigendum to "Leucine–glycine and carnosine dipeptides prevent diabetes induced by multiple low-doses of streptozotocin in an experimental model of adult mice"** **791**
